# Aircraft events correspond with vocal behavior in a passerine

**DOI:** 10.1038/s41598-020-80380-4

**Published:** 2021-01-13

**Authors:** Allison S. Injaian, Ethan D. Lane, Holger Klinck

**Affiliations:** 1 Center for Conservation Bioacoustics, Cornell Lab of Ornithology, Ithaca, NY 14850 USA; 2 Department of Ecology and Evolutionary Biology, Cornell University, Ithaca, NY 14850 USA; 3 Present Address: Odum School of Ecology, University of Georgia, 140 E. Green St., Athens, GA 30602 USA

**Keywords:** Urban ecology, Behavioural ecology, Conservation biology, Environmental impact

## Abstract

Airports can affect birds by hindering acoustic communication. Here, we investigated the impacts of aircraft events on vocal behavior in wood thrush (*Hylocichla mustelina*) breeding one mile from an airport in Ithaca, NY, USA. We identified the number of wood thrush songs between 0500 and 0800 h at various distances from the airport and on days with various morning flight schedules. We also analyzed the number of sites from which birds sang during the peak of aircraft events (proxy of number of wood thrush). We found that birds sang more from 0600 to 0640 h when there were aircraft events during this period. This increased vocal behavior is likely explained by increased song output per individual wood thrush, rather than more wood thrush vocalizing. Increased song rate may negatively affect wood thrush fitness through increased energetic demands and/or time tradeoffs with other important behaviors, such as foraging. Identifying the noise thresholds associated with fitness costs (if any) and how different behavioral strategies (i.e. changing the pattern of vocalizations) may allow individuals to evade these costs would be useful for establishing conservation policy in breeding habitats used by passerines, such as the wood thrush.

## Introduction

Transportation networks continue to expand to accommodate the growing human population, causing anthropogenic noise to affect land area across the urban–rural gradient ^1^. Indeed, anthropogenic noise, such as noise from aircraft takeoffs, landings and flyovers, has even been found to double noise levels in the majority of protected areas in the United States ^2^. Noise profiles produced by aircraft are complex and broadband, due to many components simultaneously producing sound at both high and low frequencies (e.g., engine noise and airframe noise)^3,4^. Additionally, aircraft noise profiles manifest in a frequency range that is audible to many wildlife species (approx. 500–5000 Hz)^5^ and are relatively high intensity compared to other anthropogenic noise sources. Areas approximately 300 m from a 2-lane highway with relatively high traffic volume are exposed to approximately 47 dBA^6^, whereas areas approximately 300 m from a jet take-off are exposed to approximately 100 dBA^7^—a difference that is perceived by humans to be 32 times louder^8^. The response of wildlife to aircraft noise is an area of current research, and a recent meta-analysis suggests that animals respond similarly to aircraft noise, as compared to other types of anthropogenic noise^9^. Also, habitats that host breeding birds are generally closer to roadways than runways, therefore the actual received noise levels, and thus behavioral alterations, may be functionally similar between aircraft and traffic noise.

To date, there has been much research on how animals, especially birds, respond to anthropogenic noise^10,11^. These studies have found altered habitat use^12^^–^^15^, as well as altered communication, such as increased vocal behavior^16^^–^^18^, decreased vocal behavior^19^, and/or shifts in the timing of vocal behavior^20,21^ in noisy habitats. Some studies have also found that exposure to anthropogenic noise is associated with reduced reproductive success^22,23^. Species-specific responses to noise (e.g., altered timing of vocalizations) may indicate that behavioral plasticity allows some individuals to avoid negative consequences of human-induced environmental change. However, noise-induced behavioral plasticity does not always improve communication^24^ and may also create a tradeoff between communication success and mate attraction^25^.

The intermittent and relatively unpredictable nature of some sources of anthropogenic noise, like aircraft events, may increase the likelihood of negative effects on wildlife, in certain scenarios. Indeed, unpredictable traffic noise resulted in greater behavioral alterations during the breeding period in Greater sage-grouse (*Centrocercus urophasianus*), as compared to chronic noise like natural gas drilling rigs^26^. Exposure to intermittent aircraft noise and sonic booms, a phenomenon caused by aircraft traveling at speeds greater than the speed of sound in air, has also resulted in startle responses, and altered parental behaviors, growth and reproduction in domestic animals^3,27^. Therefore, the intermittent and relatively high amplitude noise associated with early morning flights (0500–0800 h) may be associated with negative impacts on individual and/or population health for birds breeding near airports or along flightpaths. These negative consequences may be driven by the overlap of early morning aircraft noise with dawn chorus, the period of time immediately surrounding dawn during which passerines display their highest rates of vocal behavior to attract mates and maintain territories^28^. In the Northern hemisphere, this early dawn chorus is most pronounced during the spring breeding season, as early singing behavior avoids singing during optimal foraging times later in the day (when temperatures are warmer^29^). Therefore, alteration to vocal behavior during dawn chorus may result in decreased pairing success or increased energetic demands^30^.

Here, we investigated the impacts of aircraft events on vocal behavior in the wood thrush (*Hylocichla mustelina*) breeding in Sapsucker Woods Sanctuary (hereafter ‘SSW’), a bird sanctuary located approximately one mile from the Ithaca Tompkins International Airport (ITH), NY, USA. We used passive acoustic monitoring techniques to assess vocal behavior in wood thrush on days that differed in the number of aircraft events. Specifically, we calculated a ‘flight score’ for each 10-min period between 0500 and 0800 h (dawn chorus). This ‘flight score’ parameter was the sum of the number of flights that occurred immediately before and during each 10-min period from 0500 to 0800 h (see methods below). No birds were directly handled during this study. The wood thrush is a suitable species in which to study anthropogenic noise impacts, as they, like other passerines, rely on song to attract mates and maintain territories. Wood thrush vocalizations span a frequency range of approximately 2–9 kHz (Fig. 4A). Therefore, wood thrush can likely hear below 2 kHz, where much of the sound energy associated with aircraft events occurs^31^.

We hypothesized a negative relationship between exposure to noise from aircraft events and wood thrush vocal behavior during dawn chorus, such that the number of wood thrush songs would decrease with greater exposure to aircraft events. We also predicted a negative relationship between the number of sites with wood thrush song (our proxy of the number of wood thrush) and aircraft events from 0550 to 0650 h, the peak of morning aircraft events at the Ithaca Tompkins Regional Airport. If our results show a negative relationship between aircraft events and vocal behavior, wood thrush may suffer reduced mate pairing success in habitats near airports or along flightpaths.

## Results

### Song rate

**Figure 1:**
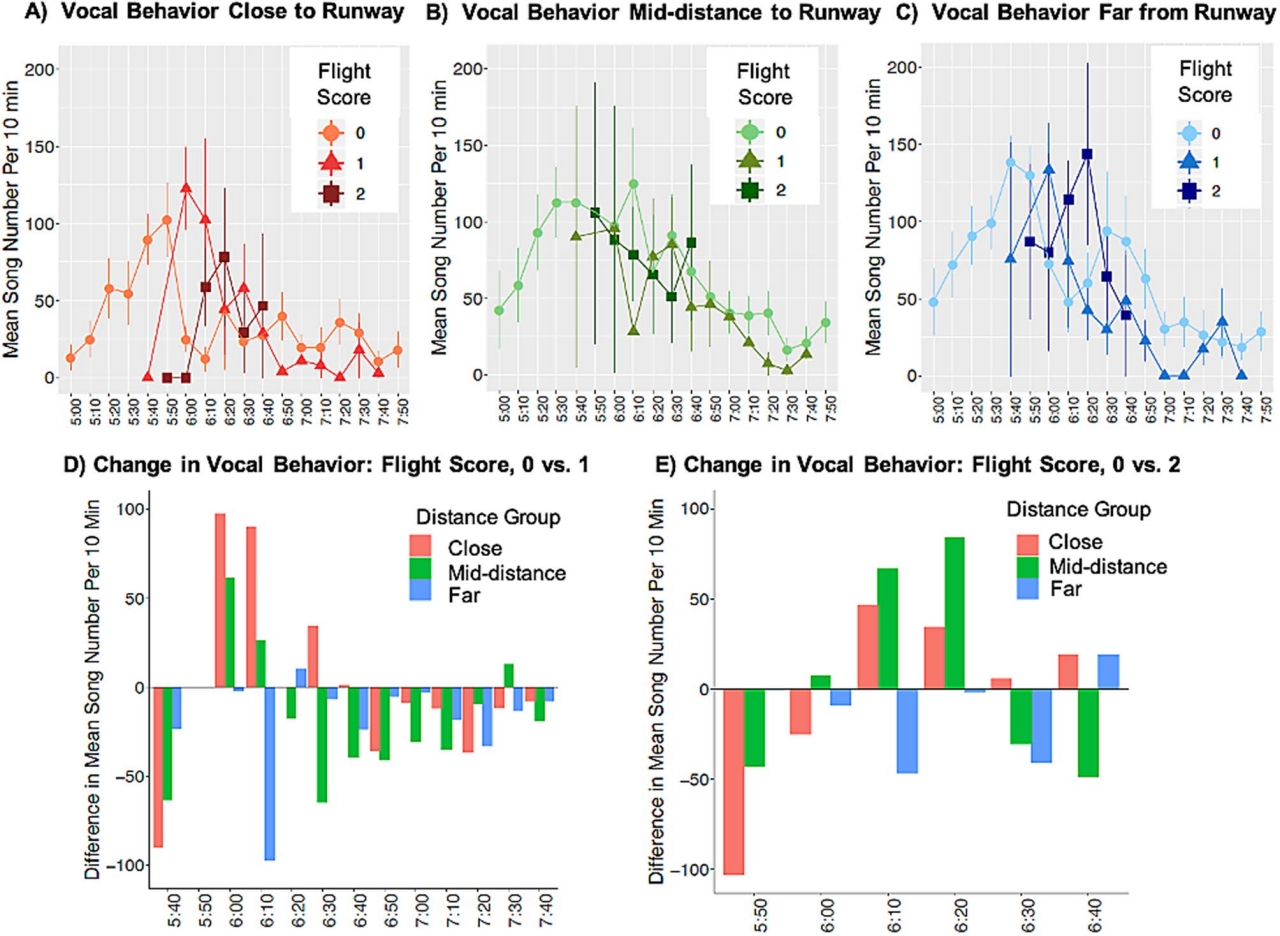
Data showing effects of aircraft events on wood thrush vocal behavior across flight scores, sites, and time. (**A–C)** Mean wood thrush song number over 10-min time periods (0500–08,000 h) for sites A) close (450–750 m) B), mid-distance (750–1050 m), and (**C**) far (1050–1350 m) from the runway. Data represent means ± 1 Standard Error, split by flight scores. Flight scores represent the number of aircraft events in the focal time period and the 10 min prior. (**D,E)** Alternative visualizations for the same data presented in (**A**–**C**). For each time period, the mean number of wood thrush songs that occurred when the flight score equaled zero was set to the baseline. The data in (**D**) and (**E**) are the differences in the mean number of wood thrush songs per 10-min time period between this baseline level and when the flight score was 1 or 2, respectively. Data are split by distance group: close (red), mid-distance (green), and far (blue).

Our results showed a positive relationship between flight score and wood thrush song output when taking into account time of day and distance from the airport. Specifically, the number of songs at sites ‘close’ to the runway was higher during the peak of aircraft activity (0550–0650 h; Fig. 1A). The best-ranked model of wood thrush vocal behavior included a three-way interaction between ‘time’, ‘flight score’ and ‘distance group’ (Table 1). Also, the 95% CIs for the ‘flight score’ parameter, as well as the interaction effect between ‘time’ and ‘flight score’, do not overlap zero (Table 2). Unsurprisingly, there was a negative relationship between ‘time’ and wood thrush vocal behavior, as peaks in wood thrush song rate occur early in the morning (Fig. 1A–C).

**Table 1:** Model comparisons for the relationship between aircraft noise and wood thrush song number.

Model*	K	AICc	ΔAICc	Weight
Time + FlightScore + Distance Group + Time*Flight Score + Time*DistanceGroup + FlightScore*Distance Group + Time*FlightScore*DistanceGroup	15	5109.34	0	0.999
Time + FlightScore + DistanceGroup + Time*FlightScore	9	5128.94	19.603	< 0.001
Time + FlightScore + DistanceGroup + FlightScore*DistanceGroup	10	5129.34	20.000	< 0.001
Time + FlightScore + DistanceGroup	8	5139.21	29.877	< 0.001
Time + DistanceGroup	7	5143.14	33.804	< 0.001
Time	5	5156.73	47.393	< 0.001

*Date and site were included in all models as random effects.

**Table 2:** Observed relationships (β estimates ± 95% CIs, calculated using ± 1.96*1 Standard Error) between response variables and parameters for the top-ranked model of wood thrush song number.

Parameter	β estimate*	95% CI
**(intercept)**	63.101	12.555, 113.655
**Time**	− 2.492	− 4.600, − 0.388
**FlightScore**	110.779	27.257, 193.852
DistanceGroup (mid)	41.068	− 17.602, 99.607
DistanceGroup (far)	52.569	− 10.155, 115.176
Time*FlightScore	− 10.138	− 18.549, − 1.668
Time*DistanceGroup (mid)	− 1.477	− 4.145, 1.1894
Time*DistanceGroup (far)	− 2.285	− 4.951, 0.402
FlightScore*DistanceGroup (mid)	− 27.490	− 130.886, 75.562
FlightScore*DistanceGroup (far)	− 84.050	− 183.926, 18.046
Time*FlightScore*DistanceGroup (mid)	2.155	− 8.102, 12.423
Time*FlightScore*DistanceGroup (far)	7.868	− 2.263, 17.752

*Bold text indicates that 95% CI did not overlap zero.

### Number of sites with song

**Figure 2:**
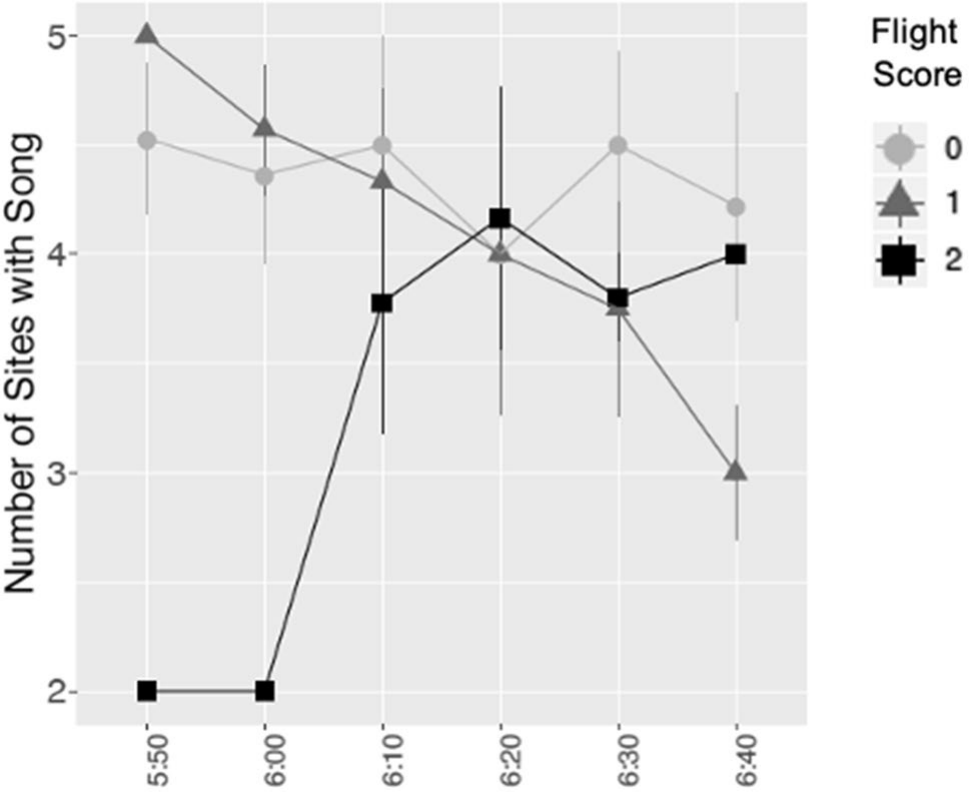
Data showing effects of aircraft events on a proxy for the number of wood thrush. The number of mean sites (± 1 Standard Error) with wood thrush songs in 10-min time periods during the peak of flight activity (0550–0650 h, n = 30 sites). Data is split by flight score (0 = light gray, 1 = medium gray, 2 = black).

The mean number of sites with wood thrush song during the peak of aircraft activity (our proxy of the number of wood thrush) did not differ based on flight score (Fig. 2). The null model was best-ranked and received approximately 75% of the model weight (Table 3).

**Table 3:** Model comparisons for the relationship between aircraft noise and habitat use in wood thrush.

Model*	K	AICc	ΔAICc	Weight
Time	3	234.02	0	0.654
Time + FlightScore	4	235.99	1.970	0.244
Time + FlightScore + Time*FlightScore	5	237.73	3.709	0.102

*Date was included in all models as a random effect.

## Discussion

Our results showed a positive relationship between aircraft events and wood thrush vocal behavior during certain time periods, which was opposite our hypothesized negative impact of aircraft events on wood thrush song. This pattern was strongest at sites closest to the runway, with a 387% average increase in wood thrush vocal behavior over baseline when flight scores equaled 1 (Fig. 1D) and a 266% average increase over baseline when flight scores equaled 2 (Fig. 1E). These results are consistent with the findings of previous studies of noise pollution on song patterns in birds^18,32,33^, as well as other taxa^17,34,35^. Additionally, natural noise sources, such as waterfalls and torrents, have been found to increase song redundancy in chaffinches (*Fringilla coelebs*)^36^. However, it is worth noting that other studies have found animals, like humpback whales (*Megaptera novaeangliae*), to be less likely to produce vocalizations given anthropogenic noise^37^. The altered vocal behavior found here may compensate for any acoustic masking of song during brief aircraft events. Alternatively, altered song may create a tradeoff between communication success and mate attraction^25^.

The fact that our data also showed no relationship between aircraft events and the number of sites with wood thrush song during the peak of aircraft activity suggests that the increase in singing activity at sites closer to the airport is not explained by stimulating more individuals to sing when flights scores equal 1 or 2, as compared to baseline. Rather, aircraft events may stimulate increased song rate within individuals. Future research investigating if noise-exposed wood thrush also alter the pitch of their song, as has been found in urban great tits^38^, and/or the complexity of their song, as has been found in red-winged blackbirds exposed to experimental noise^39^, would be of great value. Based on our data set, the timescale at which altered vocal behavior may persist in wood thrush is also unclear. However, past studies have found that other passerines return to baseline song frequency^40^ and timing patterns^41^ relatively soon after experimental noise exposure. Additionally, American black ducks (*Anas rubripes*) and European seabass (*Dicentrarchus labrax*) have been found to behaviorally habituate to variable sound impulses^42,43^. The intermittent and somewhat unpredictable nature of aircraft events at the Ithaca Tompkins International Airport (due to inconsistencies in flight schedules) may make the likelihood of habituation to aircraft noise relatively low for wood thrush in our study system.

Again, because habitat quality for wood thrush differs throughout SSW, regardless of distance from the airport, we cannot make general claims about the effects of aircraft noise on the distribution of wood thrush throughout SSW. Yet, our data suggest that forested sites that are only 450 m from the runway are still used by breeding wood thrush. This lack of avoidance of noise-exposed habitats has also been found in other passerines, such as serins^32^. However, it is worth noting that other studies have found that birds avoid noise-exposed habitats, both during breeding^14,44^ and migration^45^. It is possible that aircraft noise levels above a certain threshold (which was not reached in this study) could result in reduced wood thrush vocal behavior and/or habitat use (i.e. a dose response). Dose–response models in killer whales (*Orcinus orca*) show avoidance behavior when exposure to ship noise is greater than 150 dB re 1 μPa^46^. Also, zebra finches (*Taeniopygia guttata*) increase the amplitude of their song with increasing background noise levels, but cease to sing once background noise levels reached 80 dB^47^. Finding a noise threshold at which behavioral changes, and perhaps negative fitness impacts, are expected in wood thrush, would be highly useful in this system and are an area of future focus.

The potential conservation implications for increased vocal behavior during time periods of increased aircraft noise are unclear. The wood thrush is a near-threatened species on the *IUCN* ‘red list’, and their population declines are thought to be related to reduced availability of forested breeding habitats^48,49^. Shifts in vocalizations by birds experiencing anthropogenic noise have been associated with positive, negative, or no effects on fitness^25,50^^–^^52^. Here, it is possible that the increased wood thrush song rate is associated with slightly increased energetic demands^53,54^ and/or time tradeoffs with other important behaviors, such as foraging^55^. Alternatively, wood thrush may suffer no negative fitness consequences given increased vocal behavior, as the number of flights, and thus the time period during which vocalizations may be masked, is relatively low for the Ithaca Tompkins International Airport. Overall, the implications of increased vocalizations are unknown in this system. Although logistically difficult, future work investigating the actual fitness costs associated with altered vocal strategies for wood thrush in noise-exposed habitats would be of high value, and may be applicable across noise type^9^. Additionally, knowing the noise thresholds associated with fitness costs (if any) and how different behavioral strategies (i.e. changing the pattern of vocalizations) may allow individuals to evade these costs would be useful for establishing conservation policy in breeding habitats used by passerines, such as wood thrush.

## Methods

### Sound recordings

**Figure 3:**
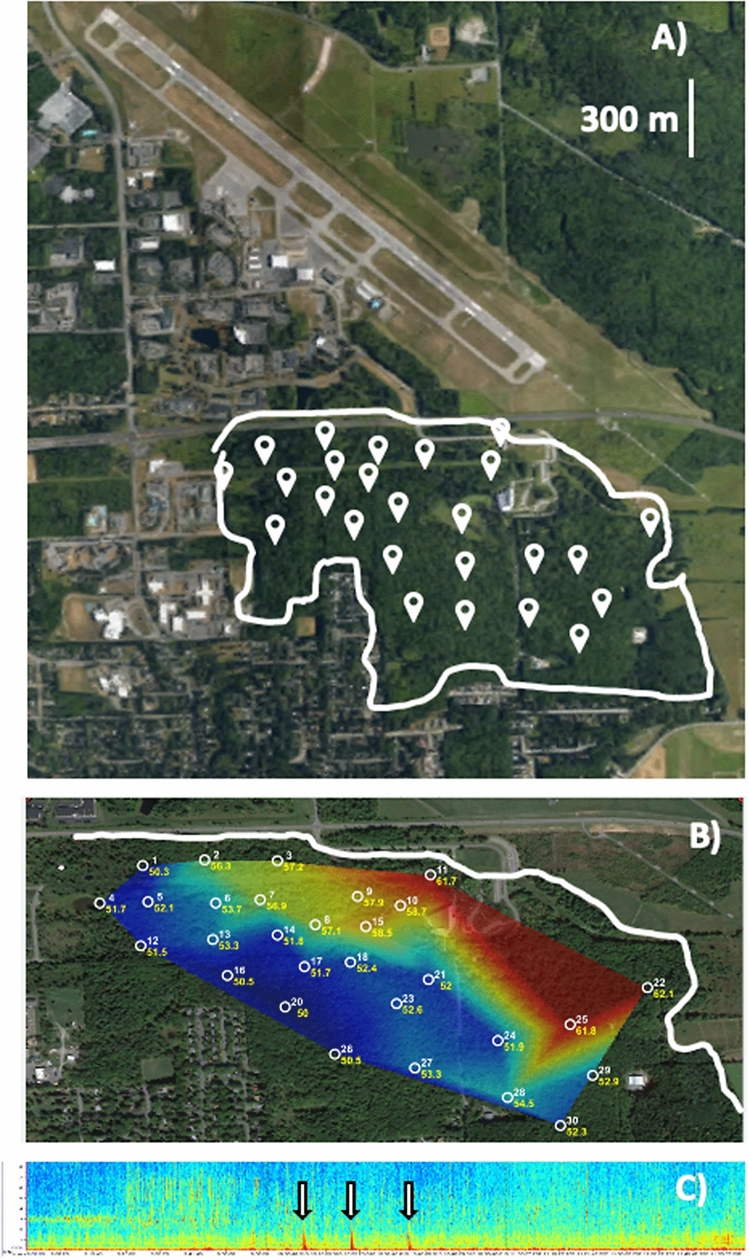
Maps and spectrograms associated with recoding sites. (**A**) Aerial map of the Ithaca Tompkins International Airport and Sapsucker Woods (outlined in white, with recording locations marked by white points), (**B**) Distribution of peak amplitude levels (dBA) associated with an aircraft overflight across recording sites in Sapsucker Woods (greater received amplitude levels indicated in red, lower received amplitude levels indicated in blue), (**C**) Spectrogram (kHz vs. h) from 0500 to 0800 h at one recording site in SSW. Aircraft overflights indicated by arrows. Satellite images for A) and B) were generated from GoogleEarth (version 7.3.2, https://earth.google.com/web/search/Sapsucker+Woods,+Ithaca,+NY/).

Recordings were taken at 30 sites in SSW (Ithaca, NY, USA; *Lat*: 42.481673, *Long*: − 76*.*460053, Fig. 3A), using autonomous recording units (*Swifts,* Center for Conservation Bioacoustics, Cornell University) mounted to trees, approximately 2 m off the ground and spaced at least 250 m apart. *Swifts* recorded in a single channel (mono) from 1 to 19 May 2017, from 0500 to 0800 h at a 48 kHz sampling rate, with a 16*-*bit resolution. The sensitivity of the omnidirectional microphone was − 44 dB re 1 V/Pa (± 2 dB) and the frequency response is flat (± 3 dB) in the relevant frequency range 100 Hz to 10,000 Hz. The gain was set to 33 dB and the clipping level of the analog to digital converter (ADC) is ± 0.9 V. *Swift* sites varied in their distance from the airport runway (456–1322 m), resulting in differential exposure to aircraft noise (Fig. 3B). Daily weather conditions were similar in Ithaca, NY, USA from 1 May to 19 May 2017 (i.e. no days in which rain functionally altered wood thrush song).

Sites were categorized as ‘close’ (450–750 m, n = 7 sites), ‘mid-distance’ (750–1050 m, n = 16 sites), or ‘far’ (1050–1350 m, n = 7 sites) from the Ithaca Tompkins International Airport runway for analysis. These three distance categories were chosen because they equally split the range of distances (456–1322 m) from the runway that existed in our data and created a parameter across which peak exposure to aircraft noise differed between groups (ex. 5 May 2017: ‘close’; 73.8 dBA ± 3.9 SD, ‘mid-distance’; 69.3 dBA ± 5.2 SD, ‘far’; 67.3 dBA ± 5.5 SD). Distance from the runway was measured from the GPS coordinates of each recording location (in decimal degrees, accurate to approximately ± 3 m) and the center of the only runway at Ithaca Tompkins International Airport. Additionally, habitat type varies throughout SSW, with larger, denser portions of forest located further from the airport (see Fig. S2). Because wood thrush prefer these larger, denser forests, and thus might be more likely to colonize sites further from the airport for reasons unrelated to aircraft noise, we focused on within distance-group variation for analysis of vocal behavior.

We used a band-limited energy detector in *Raven Pro 2.0* (Center for Conservation Bioacoustics, Cornell Lab of Ornithology) to identify all flight times between 0500 and 0800 h on focal days (minimum frequency = 200 Hz, maximum frequency = 1000 Hz, minimum duration = 10 s, maximum duration = 45 s, minimum separation = 60 s, minimum occupancy = 30%, SNR = above 20 dB, block size = 180 s, hop size = 90 s, percentile = 20%). The detector output was then manually checked by researchers who listened to each auto-detection to ensure it was an aircraft event. The ambient noise levels (L_10_ dBA) in SSW were calculated from 0600 to 0700 h on a subset of days in May 2017 (see supplement). Based on these data, ambient noise levels in SSW differed during time periods associated with aircraft overflights (*5 May:* 69.1 L_A10_ ± 7.2 SD, *9 May:* 60.9 L_A10_ ± 1.9 SD, *19 May*: 65.8 L_A10_ ± 4.8 SD), as compared to time periods without overflights (*5 May:* 58.5 L_A10_ ± 2.5 SD, *9 May:* 54.5 L_A10_ ± 2.4 SD, *19 May*: 58.2 L_A10_ ± 3.6 SD, see supplement for more details). Given that an increase of 10 dBA is perceived as twice as loud in humans^8^ and passerines’ perceptions of sound is thought to be similar, but slightly less sensitive, than humans^31^, our data suggest that aircraft events have a substantial impact on the soundscape for birds breeding in SSW (Fig. 3C and Supplementary Fig. S1). Also, aircraft events altered the acoustic signature of SSW for an average of 47.5 s (± 27.4 s SD), similar to previous studies of aircraft noise profiles^56^.

### Sound analysis

We analyzed the number of wood thrush songs in a subset of recordings in May 2017 (n = 10 days, Mon–Fri only, May: 1, 2, 3, 4, 5, 8, 9, 15, 17, 18). These specific dates were chosen because they fell within the early breeding period for wood thrush in Ithaca, NY, USA, when singing for mate attraction and territory establishment is most common. These dates also varied in their flight schedule and time of first flight, thus allowing us to control for the potential impacts of time of day, time of first flight, and number of flights on wood thrush vocal behavior. On each focal day, we identified all sites with wood thrush song from 0500 to 0800 h and used data from these sites for our analyses.

To decrease the likelihood of duplicate song records from a given individual in our analysis, we eliminated sites with simultaneous song if the sites were less than 500 m apart (i.e. adjacent *Swift* units). Given that we do not have field data on the number of wood thrush territories present in SSW during May 2017, the presence of distinct wood thrush song across recording sites is our best estimate of the number of wood thrush singing in any given 10-min time period. It is important to acknowledge that wood thrush territory size ranges from 0.08 to 4 ha (approximately 800–40,000 m^2^). Therefore, we may have had multiple wood thrushes recorded by one unit, or multiple recording units within a single wood thrush territory. Nonetheless, this limitation should not impact our ability to identify relationships between aircraft events and vocal behavior, as we focused our analysis on changes in vocal behavior compared to a baseline level (flight score = 0; *see below for details*). Therefore, the spatial data found here can provide useful information on the relationship between aircraft events and wood thrush vocal behavior within SSW and across time.

**Figure 4:**
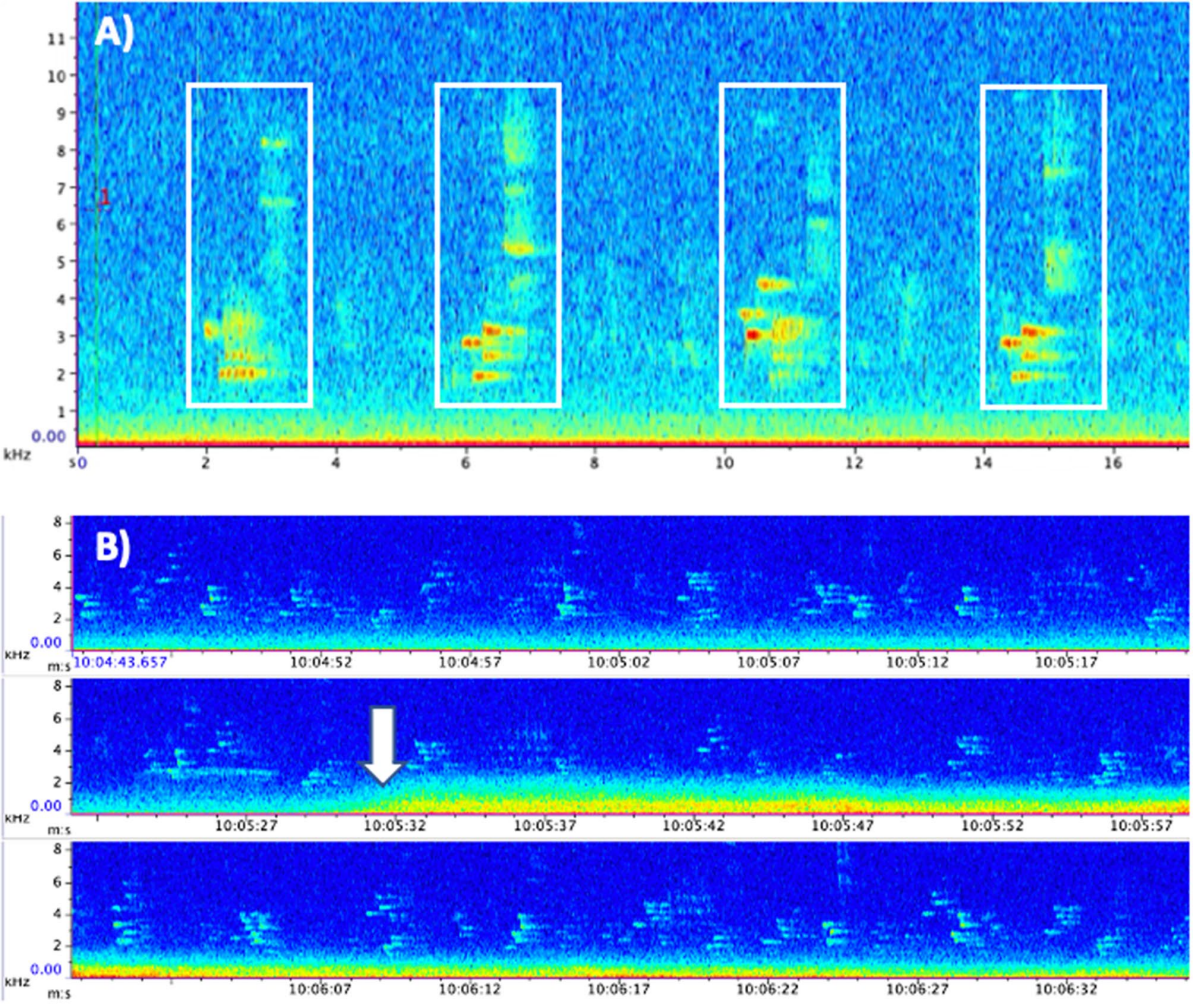
Spectrograms associated with recoding sites. (**A**) Spectrogram (kHz vs. s) of four wood thrush songs, with each song boxed in white. (**B**) Spectrogram (kHz vs. s) of wood thrush songs before, during and after an aircraft event (start of aircraft event indicated by the white arrow).

We used *Raven Pro 2.0* to visualize sound recordings and identify all sites with wood thrush song before, during, and after aircraft events between 0500 and 0800 h (Fig. 4B). Researchers trained in wood thrush song identification used template detectors in *Raven Pro 2.0* (detection frequency range = 200 Hz and threshold = 0.6–0.8 depending on quality of recording, with loose matching and non-merged templates) to detect and annotate each individual wood thrush song (Fig. 4A). Detector outputs were also manually checked to ensure accuracy.

In addition, we analyzed recordings to assess the relationship between aircraft events and the number of sites with wood thrush song (our proxy of the number of wood thrush). Specifically, we focused on the peak of morning aircraft activity at the Ithaca Tompkins International Airport, which occurs from 0550 to 0650 h. These data on the number of sites with wood thrush song allow us to contextualize any differences in song number. For example, if we found a negative relationship between aircraft events and wood thrush song number, but no difference in the number of sites with wood thrush song, our results would suggest that wood thrush are reducing song rate to some non-zero value, but not leaving their territories or completely ceasing song production during time periods affected by aircraft noise.

To assess whether sites had thrush territories present, we again used *Raven Pro 2.0* to visualize sound recordings from 0550 to 0650 h, the peak of morning aircraft activity at our sites. Therefore, our data represented the presence or absence of wood thrush song in each 10-min time period at each site during May 2017. We then compared the number of sites with wood thrush song in each 10-min time period across flight scores (flight score = 0, 1, or 2; see paragraph on statistical analysis below). Similar to our previously described methods, we only included simultaneous song data if the sites were greater than 500 m apart to avoid duplicate bird song in our analysis. Unfortunately, there were no days in May 2017 in which zero flights went out from the Ithaca Tompkins International Airport between 0550 and 0650 h; therefore, we cannot gauge wood thrush vocal behavior in the complete absence of aircraft events.

**Figure 5:**
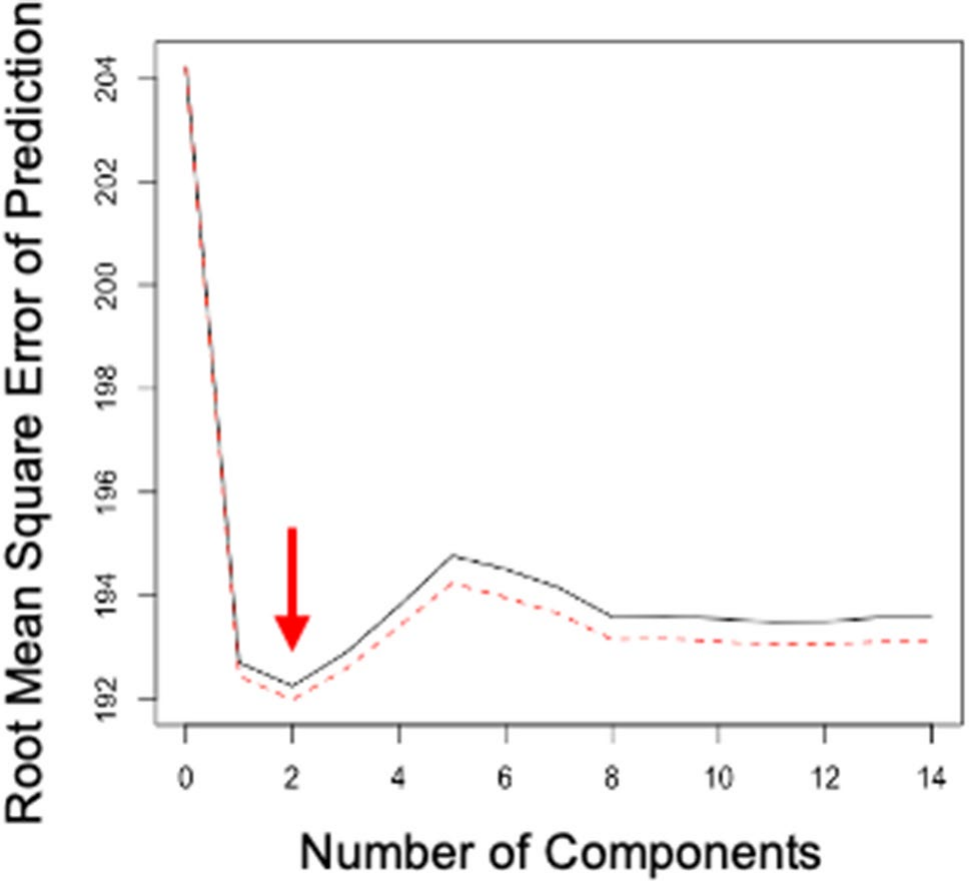
Figure output from partial least squares analysis. Root mean square error of prediction (RMSEP) shown across ‘flight impact score’ components (e.g. FlightNumber_T_ + FlightNumber_T−1_, + FlightNumber_T−2_ … FlightNumber_T−13_, n = 14 components). The red arrow indicates that the lowest RMSEP is achieved with 2 components.

To better understand the relationship between aircraft noise and wood thrush vocal behavior across time, we summed the number of songs per 10-min time period at each site, from 0500 to 0800 h. Wood thrush vocal behavior during a focal 10-min time period may be affected by flight(s) that occurred in that time period, as well as any flights that occurred earlier in the morning. To identify the extent to which flight history affects wood thrush songs in any successive 10-min time period, we ran a partial least squares analysis which included a flight number parameter for each focal 10-min time period (e.g. FlightNumber_T_ + FlightNumber_T−1,_ + FlightNumber_T−2_… FlightNumber_T−13_, n = 14). The partial least squares analysis indicated that the error was lowest with the first two parameters (e.g. FlightNumber_T+_ FlightNumber_T−1_, Fig. 5). Therefore, we created a ‘flight score’ parameter that was the sum of the number of flights that occurred in the focal 10-min time period, as well as the 10-min time period prior (i.e. if the focal time period was 0600–0610 h and there was one flight at 0552 and another flight at 0607, the flight score associated with the focal time period would be 2). We then ran generalized linear models, with the number of wood thrush songs per 10-min time period as the dependent variable. In each model in our model set, we included ‘site’ and ‘date’ as random effects, and ‘time’ as a fixed effect. We included ‘date’ in these analyses to account for the change in sunrise (and thus the expected time at which birds will start singing) and the reproductive stage of individuals. Additional models included a combination of the main effects and/or interaction effects for ‘distance group’ and ‘flight score’ parameters. Both ‘distance group’ and ‘flight score’ were included as fixed effects (see Table 2 for the full model set).

For our second analysis, data recorded the presence or absence of wood thrush song in each 10-min time period, across sites and days. Therefore, we ran generalized linear mixed models with binomial distributions, using the *glmer* function in the *glmm* package in R. All models in this model set included ‘date’ as a random effect. We also included models that accounted for both the main and/or interaction effects between ‘time’ and ‘flight score’ (see Table 1 for the full model set).

For the models associated with each analysis, we used the values of Akaike Information Criterion corrected for small sample sizes (AICc) and model weights for model comparisons^57,58^. These comparisons allowed us to evaluate the hypotheses that exposure to aircraft events was related to our dependent variables: if models including the measure of ‘flight score’ were ranked higher than models that did not include this term, our results suggest that aircraft events explain a considerable proportion of variation in our data. For the top-ranked models that included the ‘flight score’ parameter, we estimated the effect size (β parameter estimates) and 95% confidence intervals (CI) of our noise parameters through the *summary* function in R. We assessed the importance of effect sizes based on whether the 95% CI overlapped zero (Table 2).

## Supplementary Information

Supplementary file.

## Data Availability

Data will be made available on Dryad upon acceptance.
