# Supplementary material for: Aircraft events correspond with vocal behavior in a passerine
Source: Sci Rep. 2021 Jan 13;11:1197. doi: 10.1038/s41598-020-80380-4 (PMC7806583; doi:10.1038/s41598-020-80380-4)
Supplement: Supplementary file 1 — Supplementary file. [file 41598_2020_80380_MOESM1_ESM.docx]

Aircraft events correspond with vocal behavior in a passerine

Allison S. Injaian^1,2,a,*^, Ethan D. Lane^2^, Holger Klinck^1^

^1^Center for Conservation Bioacoustics, Cornell Lab of Ornithology, Ithaca, NY, USA 14850

^2^Department of Ecology and Evolutionary Biology, Cornell University, Ithaca, NY, USA, 14850

^a^Current address: Odum School of Ecology, University of Georgia, Athens, GA, USA, 30602

* Corresponding author:

Allison S. Injaian

140 E. Green St.

Athens, GA 30602

[inja@uga.edu](mailto:inja@uga.edu)

*Determining ambient noise levels*

We determined ambient noise levels in Sapsucker Woods (SSW, Ithaca, NY, USA) during time periods associated with and without aircraft events from 0600 to 0700 h on 5, 9, and 19 May 2017, to ensure that aircraft events did indeed alter the acoustic landscape for birds breeding in SSW (Fig. S1A-C). First, we used a *Matlab* ‘peakfinder’ function to identify the timing of the aircraft events. Then, we calculated the peak levels associated with aircraft events (L_A,Max_) from the A-weighted 20Hz to 20kHz broadband levels, at a 1-sec time resolution for a window - 15 s to + 20 s around the aircraft event time. Next, we calculated the L_10_ values, which indicate the amplitude level which was exceeded 10% of the time during the measurement period, for the same time window around each aircraft event (- 15 s to + 20 s) from 0600 to 0700 h using a 1-sec time resolution. Finally, we compared the average L_10_ values for 1-min time intervals that included or excluded aircraft events. This comparison allowed us to assess if the sound levels in SSW differed during time periods associated with aircraft events, as compared to time periods not associated with aircraft events.

1. May 5, 2017


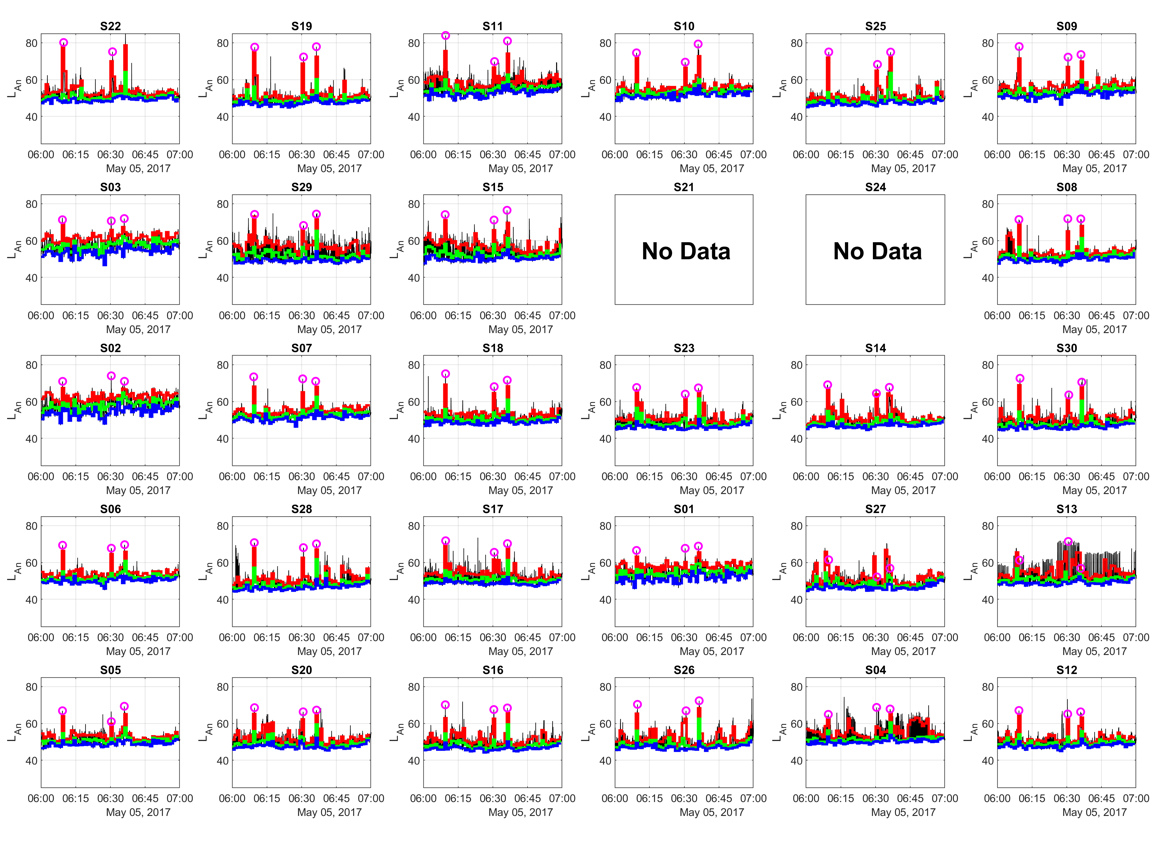


1. May 9, 2017


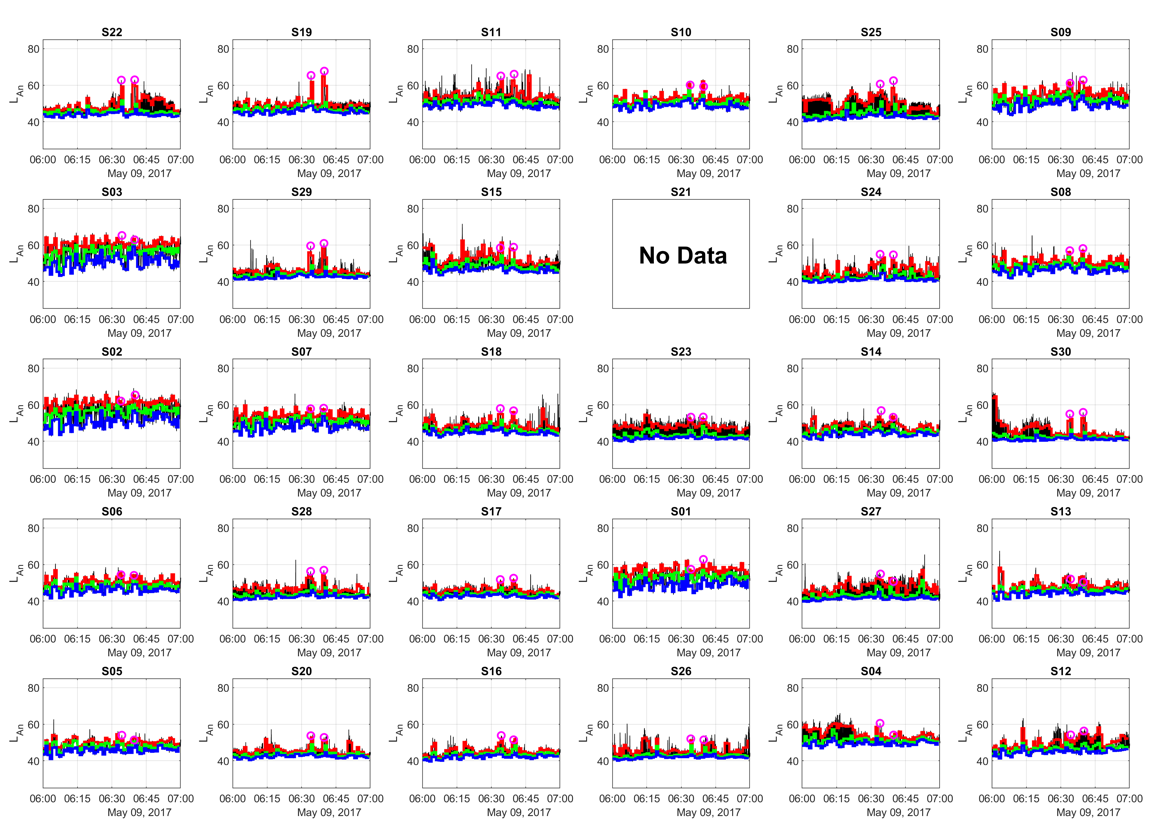


1. May 19, 2017


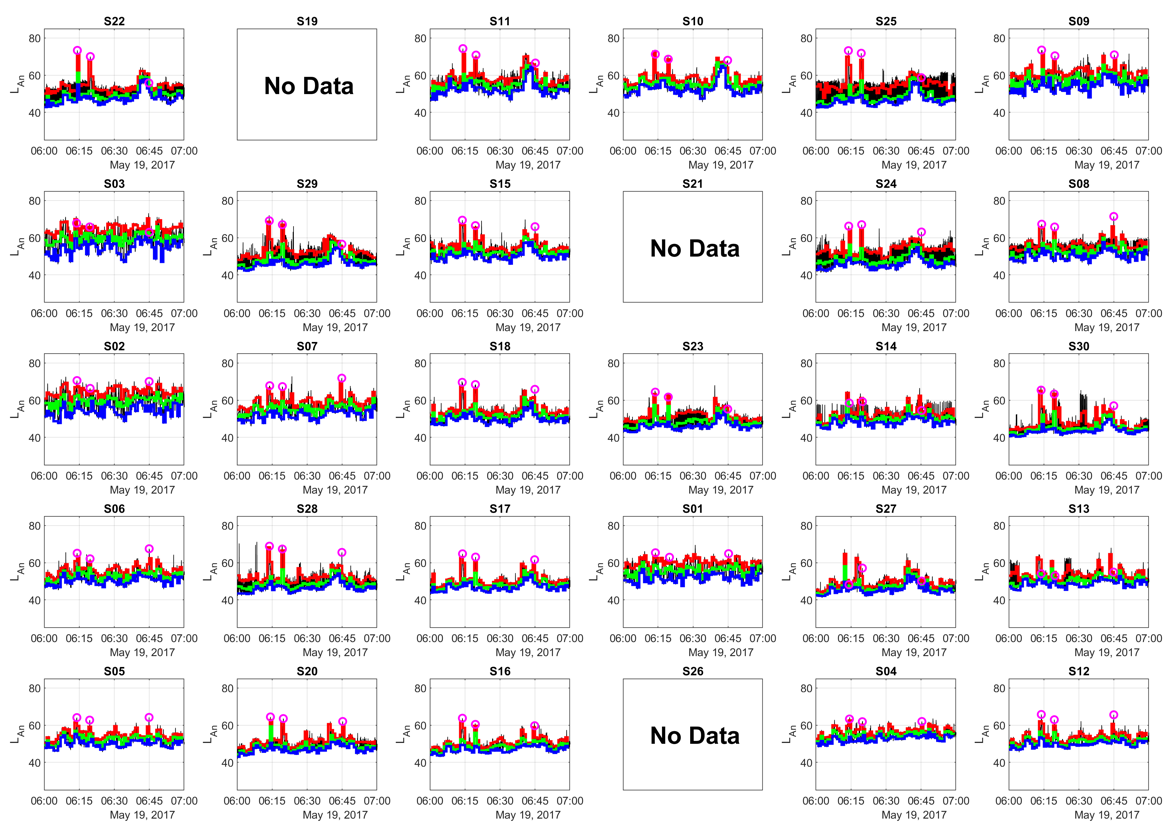


Fig. S1. Data represent ambient sound levels (dBA) from each of the 30 recording sites (S01 – S30) in Sapsucker Woods Bird Sanctuary in Ithaca, NY, USA on A) 5 May 2017, B) 9 May 2017 and C) 19 May 2017. The peak levels associated with aircraft events (L_Amax,1sec_) are represented by magenta circles. The percentiles L_A10,_ L_A50_, and L_A90_ are represented by red, green, and blue lines, respectively. The 1-sec broadband levels are represented by the black line at the back of each panel.


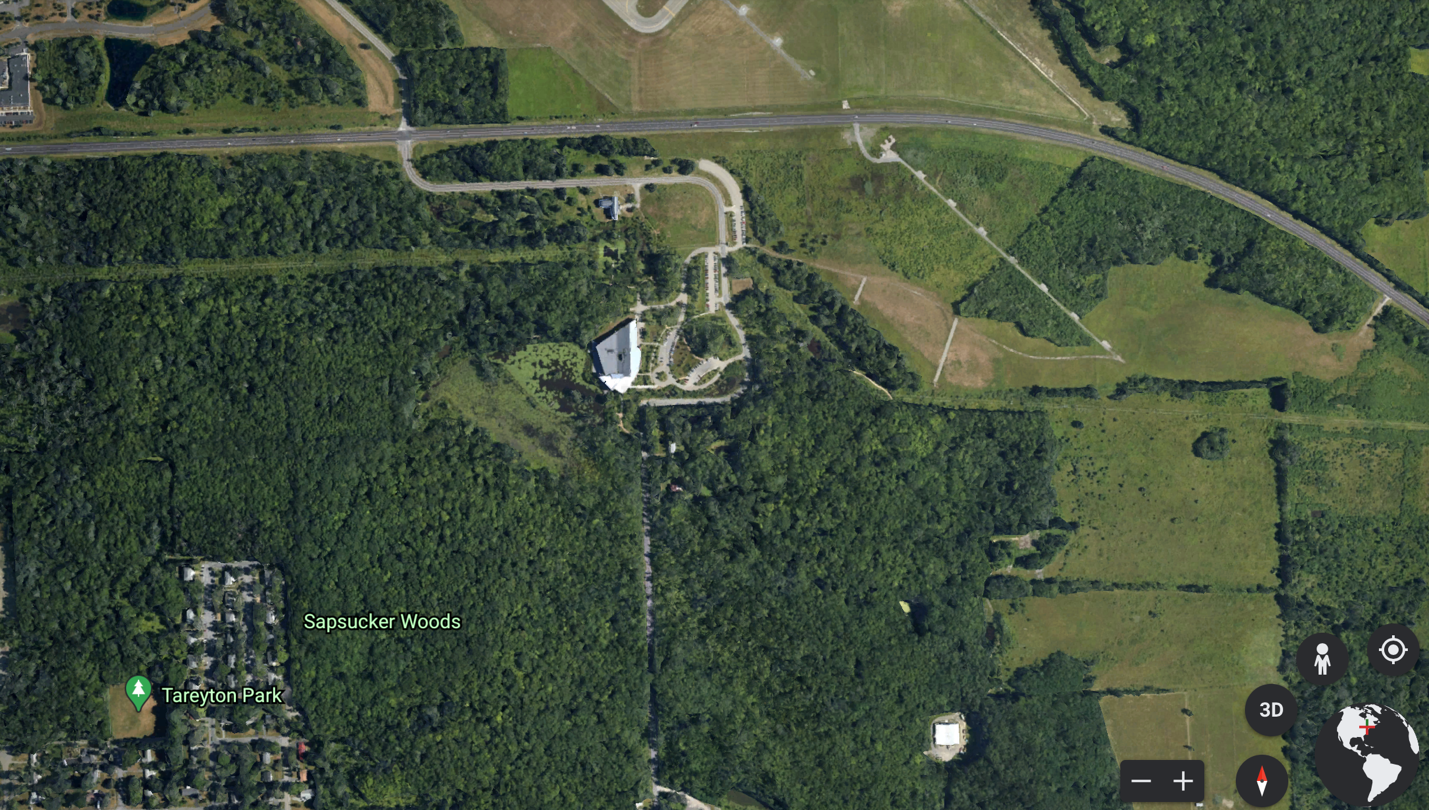


**Ithaca Tompkins International Airport**


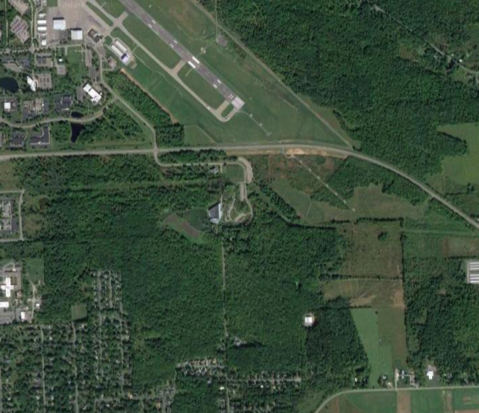


**A)**

**B)**

Figure S2. A) Aerial view of and Sapsucker Woods and Ithaca Tompkins International Airport. B) Close up version of aerial view that highlights the less dense forested potion of Sapsucker Woods located closer to Ithaca Tompkins International Airport (white, dashed oval), as compared to the more densely forested portion of Sapsucker Woods that is further from Ithaca Tompkins Regional Airport (white, solid oval). Satellite images were generated from GoogleEarth (version 7.3.2, <https://earth.google.com/web/search/Sapsucker+Woods,+Ithaca,+NY/>)
